# Dobutamine stress MRI catheterisation in patients with hypoplastic left heart syndrome after Fontan completion: preliminary results

**DOI:** 10.1186/1532-429X-13-S1-P212

**Published:** 2011-02-02

**Authors:** Hannah R Bellsham-Revell, Aphrodite Tzifa, Tarique Hussain, Isra Valverde, Aaron Bell, Philipp Beerbaum, John M Simpson, Victoria Parish, Shelby Kutty, Reza Razavi, Gerald Greil

**Affiliations:** 1 King's College London, London, UK; 2 Guy's and St Thomas' Foundation NHS Trust, London, UK; 3 University of Nebraska/Creighton University Joint Division of Pediatric Cardiology, Omaha, NE, USA

## Introduction

Exercise tolerance is often impaired in children with hypoplastic left heart syndrome(HLHS). It has been hypothesised this may be related to diastolic dysfunction of the single systemic right ventricle (RV), ventricular hypertrophy or to restricted pre-load from limited pulmonary venous return in Fontan physiology.

## Methods

Children with HLHS referred for dobutamine stress MRI catheterisation were assessed with MRI volumetry and invasive pressure measurements at rest and at 10mcg/kg/min and 20mcg/kg/min of dobutamine. Results were normalised for body surface area and compared with normal adult left ventricle (LV) and RV^1^.

## Results

**Figure 1:**
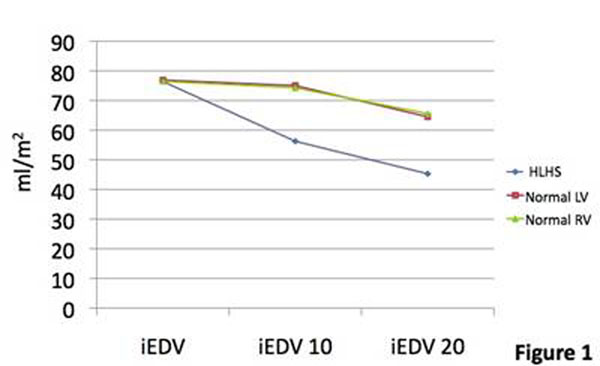


**Figure 2:**
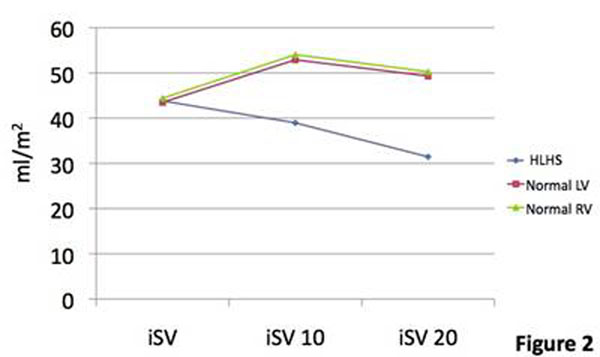


**Figure 3:**
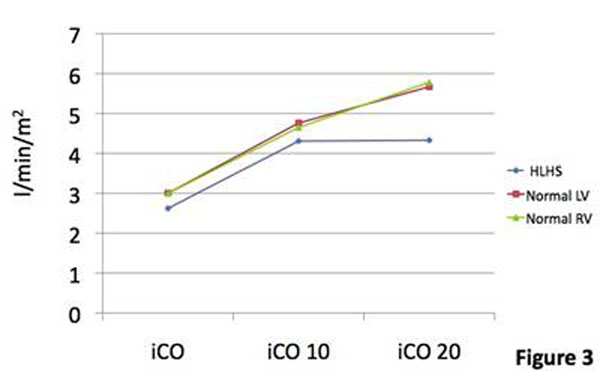


**Figure 4:**
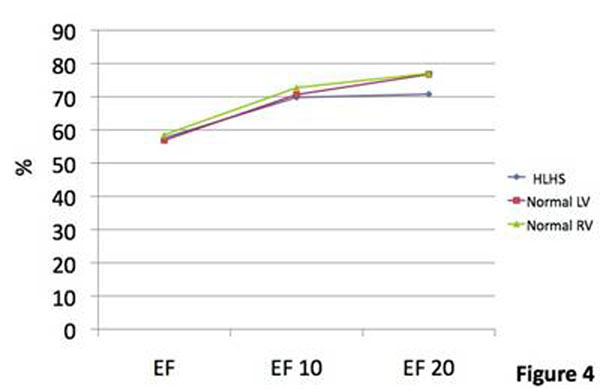


Four patients were studied (indication was decreased exercise tolerance). Median (range) age was 9.2 years(5.9-11.6). Heart rate (HR) increased at both levels of stress: mean (standard deviation) at rest 60(±7.0), 10mcg/kg/min 110.8(±13.7), 20mcg/kg/min 137.5(±7.0). The rate of increase between rest and 10mcg/kg/min appeared higher than in the normal group. Indexed end diastolic volume (iEDV) fell considerably more between rest and 10mcg/kg/min compared to both the normal LV and RV(figure 1). Indexed end systolic volume (iESV) fell similarly to the normal LV and RV. The steeper fall in iEDV led to a decreased indexed stroke volume (iSV) at the first level of stress in contrast to an elevation in iSV in both the normal ventricles. In both groups there was a fall in iSV between the first and second levels of stress (figure 2). Indexed cardiac output (iCO) rose similarly in both groups, but there was no further rise in the HLHS group between the first and second level of stress. This suggested that the HLHS group at stress are reliant on increased HR to raise iCO (figure 3), but that at 20mcg/kg/min despite an increase in HR, it is not sufficient to increase the iCO. Ejection fraction increased at 10mcg/kg/min in both groups, but with little further change in the HLHS (both normal ventricles increased further at 20mcg/kg/min) (figure 4). Changes in lateral tunnel pressure and RV end diastolic pressure were inconsistent between the patients and warrant further study. All children tolerated both levels of stress. Dobutamine did not need to be discontinued in any patients, nor were any arrhythmias observed

## Conclusion

Dobutamine stress MRI in HLHS patients after Fontan completion appears to be safe and feasible, may provide insights into the physiology of the systemic RV at stress, and can potentially contribute to the understanding of decreased exercise tolerance in this group.

